# Anti-proliferative effect of melatonin in human hepatoma HepG2 cells occurs mainly through cell cycle arrest and inflammation inhibition

**DOI:** 10.1038/s41598-023-31443-9

**Published:** 2023-03-16

**Authors:** Heba K. Nabih, Ahmed R. Hamed, Shaymaa M. M. Yahya

**Affiliations:** 1 Medical Biochemistry Department, Medicine and Clinical Studies Research Institute, National Research Centre, 33 El Bohouth St., Dokki, Giza, 12622 Egypt; 2 Chemistry of Medicinal Plants Department, and Biology Unit, Central Laboratory for Pharmaceutical and Drug Industries Research Institute, National Research Centre, 33 El Bohouth St., Dokki, Giza, 12622 Egypt; 3 Hormones Department, Medicine and Clinical Studies Research Institute, and Stem Cell Lab, Centre of Excellence for Advanced Sciences, National Research Centre, 33 El Bohouth St., Dokki, Giza, 12622 Egypt

**Keywords:** Biochemistry, Molecular biology

## Abstract

Hepatocellular carcinoma (HCC) is the major lethal primary liver malignant worldwide. Although, melatonin has various antitumor bioactivities; there is a requirement for more investigations to elucidate the not discussed effects, and the controversial responses of the treatment with melatonin on targets mediated in HCC. To achieve the aim of the present study, HCC-HepG2 cells were treated with different concentrations of melatonin at various time intervals. The selected minimal proliferation inhibition doses of melatonin were then incubated with cells to examine the arresting effect of melatonin on dividing cells using flow cytometry. Furthermore, the molecular patterns of genes that contributed to apoptosis, drug resistance development, antioxidation, and melatonin crossing were quantified by qRT-PCR. Additionally, the Human inflammation antibody array membrane (40 targets) was used to check the anti-inflammatory effect of melatonin. Our results validated that, melatonin shows anti-proliferative action through preserving cells in G0/G1 phase (*P* < 0.001) that is associated with a highly significant increase in the expression level of the P53 gene (*P* < 0.01). On contrary, as a novelty, our data recorded decreases in expression levels of genes involved in the pro-apoptotic pathway; with a significant increase (*P* < 0.05) in the expression level of an anti-apoptotic gene, Bcl2. Interestingly, we detected observed increases in the expression levels of genes responsible for conferring drug resistance including ABCB1, ABCC1, and ABCC5. Our study proved the anti-inflammatory activity of 1 mM melatonin in HCC-HepG2 cells. Accordingly, we can conclude that melatonin facilitates the anti-proliferation of cells at doses of 1 mM, and 2.5 mM after 24 h. This action is initiated through cell cycle arrest at G0/G1 phase via increasing the expression of P53, but independently on apoptosis. Collectively, melatonin is an effective anti-inflammatory and anti-proliferative promising therapy for the treatment of HCC. However, its consumption should be cautious to avoid the development of drug resistance and provide a better treatment strategy.

## Introduction

Hepatocellular carcinoma (HCC) is the most common cancer among all primary liver cancers, accounting for more than 70% of cases, and be the third leading cause of cancer-related death in 2020, with expected increased incidences, and deaths each year, as predicted by the World Health Organization^1^. As HCC is usually late diagnosed at advanced stages, the effective treatment options are so limited. Thus, searching for effective and safe antitumor treatments is an essential requirement^2^. Moreover, the selection of specific drugs which attenuate the cancer growth mechanisms, including cell division acceleration via suppressing cell cycle proliferation, has emerged as a promising need for developing treatments for cancers^3,4^. Besides, it has been reported that HCC cells can confer resistance to chemotherapeutic drugs^5^, and this resistance may exist before treatment (intrinsic) or develop after therapy (acquired). The different mechanisms of drug resistance were discussed in many works of literature^6,7^, in particular, which depend on ATP-binding cassette transporters (ABCs). Therefore, the complexity of drug resistance development suggests that antitumor therapies which should consider ATP might provide better strategies and improved efficacy for combating drug resistance in cancer^8^.

Melatonin (N-acetyl-5-methoxytryptamine) is an indoleamine neurohormone that is naturally produced principally by the pineal gland, as well as the liver, from the amino acid tryptophan. Nevertheless, its secretion was found to be decreased in the elderly, so many populations consume melatonin as a supplement that regulates circadian rhythmicity at night. Melatonin works through independent and dependent ways of binding to G protein-coupled receptors MT1 and MT2^9^ in human cells, including hepatocytes, upon their expression induction by melatonin administration^10^. Many in vivo and in vitro studies have illustrated the physiological and pharmacological activities of melatonin, which include inhibition of proliferation, anti-inflammation, management of cellular redox status, modulation of autophagy, regulation of apoptosis, and cell cycle. Consequently, melatonin was suggested as a safe antitumor agent, either alone or in combination with other anti-cancer drugs, which would form a novel strategy for the treatment of various cancers by affecting multiple carcinogenic signaling pathways^11,12^. Meanwhile, there are few investigations have addressed the mechanistic role of melatonin in liver carcinoma^13^; therefore, the present work aimed to elucidate the dual function and the controversial responses of the treatment with melatonin on bioactive targets contributed to the proliferation, apoptosis, inflammation, and drug resistance of HCC cells.

Since, the human HCC cell line (HepG2) was the most widely used in vitro model for studying, and performing explorations of liver cancer cell therapeutic responses, and evaluating the cytotoxicity of many effective drugs^14^, we conducted our experiments on this type of permanent cells originally derived from a well-differentiated patient with HCC.

## Material and methods

### Cells propagation

The wild HepG2 cell line was purchased from the American Type Culture Collection (Rockville, MD, United States). The cells were cultured and maintained in Dulbecco’s Modified Eagle’s media (DMEM) (Invitrogen, USA) supplemented with 10% Fetal Bovine Serum (Invitrogen, USA), 1% (v/v) sodium pyruvate, 1% l-glutamine, and 100 Units/ml penicillin–streptomycin (Invitrogen, USA). The cultured cells were kept under the conditions of humidity, and at 37 °C in a 5% CO_2_ incubator^2^.

### Cell viability assay

After preparation of different concentrations (0–10 mM) of melatonin (Sigma Aldrich, St. Louis, MO, United States) dissolved in 0.5% dimethyl sulfoxide (DMSO) (Molecular Biology Grade; Serva, Germany), the seeded HepG2 cells onto a 96-well plate at a density of 1 × 10^4^ cells/well were incubated with melatonin concentrations for a time interval of 24 h, 48 h, and 72 h. The viability of cells was measured using MTT (3-(4,5-dimethylthiazol-2-yl)-2,5-diphenyltetrazolium bromide) colorimetric assay. MTT (Serva, Germany) working solution (5 mg/ml) was added to each well and incubated for 90 min at 37 °C. After that, DMSO (100 µl/well) was added to dissolve the formazan crystals with shaking for 10 min. The optical density of each well was measured using a microplate reader (Tristart lb 942 microplate reader; Berthold, Germany) at 492 nm against a blank (no cells). Cell viability was calculated as the percentage of viable cells in the melatonin-treated cells versus the untreated control cells. The different cytotoxic concentrations of the compound that are corresponding to decreased cell viability were calculated from the cell viability (cytotoxicity) curve using fitting into a non-linear regression equation on Prism 8 (GraphPad Software Inc; San Diego, CA, United States)^15^.

### Cell cycle distribution by flow cytometry

Cell cycle distribution was analyzed by a method of DNA content staining using propidium iodide (PI). Wild HepG2 cells were grown at a density of 1 × 10^6^ cells/well in a 6-well plate, and then treated with the calculated minimal inhibition doses of 1 mM and 2.5 mM of melatonin for 24 h. Following, the cells were washed with (Dulbecco’s phosphate buffered saline) DPBS, trypsinized using 0.05% trypsin–EDTA, fixed in 60% ethanol in DPBS, and then kept at 4 °C overnight. Cells were incubated with 10 µg/ml PI (Thermo Scientific, USA) containing 50 µg/ml RNase A in the dark for 30 min at 37 °C^16^. Cells were then analyzed using an FL2 (λex/em 535/617 nm) signal detector (ACEA Novocyte™ flow cytometer, ACEA Biosciences Inc., San Diego, CA, USA). For each sample, 12,000 events are acquired. The percentage of cells in the G0/G1, S, G2/M, and sub-G1 phases of the cell cycle was calculated using ACEA NovoExpress™ software (ACEA Biosciences Inc., San Diego, CA, USA). The experiment was repeated 3 independent times.

### RT-PCR quantitative analysis of the genes contributed to apoptosis, drug efflux, anti-oxidation, and melatonin crossing

After seeding of HepG2 cells onto a 6-well plate with a density of 300,000 cells/well, cells were treated with the calculated minimal proliferation inhibition dose of melatonin (1 mM) for 24 h. Then, cells were lysed with Qiazol Reagent (Qiagen, Germany) for RNA extraction. By following the manufacturer's instructions for the SensiFAST SYBR No-ROX one-step kit (Bioline, USA), genes expression levels were amplified and quantified by qRT-PCR (MiniOpticon Real-Time PCR System, Bio-Rad, France). Briefly, RT-PCR program steps was set up as shown in Table 1, for a sample with total volume of 15 µl containing 7.5 µl 2× sensiFAST SYBR No-ROX mix, 0.3 µl Ribosafe RNase Inhibitor, 0.15 µl Reverse transcriptase, 3.85 µl H_2_O, 2 µl RNA sample, and 0.6 µl of each specific primer sequence as indicated in Table 2. All RT-PCRs were performed in duplicate, and the gene copy numbers were normalized to 100,000 copies of the housekeeping beta-actin gene^15^.

**Table 1:** qRT-PCR program 2-step cycling.

Number of cycles	Temperature	Time	Notes
1	45 °C	10 min	Reverse transcription
1	95 °C	2 min	Polymerase activation
40	95 °C	5 s	Denaturation
	60 °C	20 s	Annealing/extension

**Table 2:** Sequences of primers.

Genes	Forward	Reverse
β-actin	5′-CCTTCCTGGGCATGGAGTCCT-3′	5′-GGAGCAATGATCTTGATCTTC-3′
Caspase-3	5′-TGGTTCATCCAGTCGCTTTG-3′	5′-ATTCTGTTGCCACCTTTCGG-3′
Caspase-7	5′-GGAGAAAGCTCATGGCTGTGT-3′	5′-TCCCCTTGGCTGTGTTTTG-3′
P53	5′-TGCGTGTGGAGTATTTGGATG-3′	5′-TGGTACAGTCAGAGCCAACCTC-3′
Bcl-2	5′-GATTGTGGCCTTCTTTGAG-3′	5′-CAAACTGAGCAGAGTCTTC-3′
Bax	5′-GTTTCATCCAGGATCGAGC-3′	5′-GCCGTCAGAAAACATGTCAG -3′
ABCB1	5′-AGACATGACCAGGTA-TGCCTAT-3′	5′-AGCCTATCTCCTGTCGCATTA-3′
ABCC1	5′-CATTCAGCTCGTCTTGTCCTG-3′	5′-GGATTAGGGTCGTGGATGGTT-3′
ABCC2	5′-CCTGGAAGATGTTGAAAAGAAAA-3′	5′-CTGAAGGCAGAAAGACTGAATGA-3′
ABCC3	5′-TTTTCTGGTGGTTCACAAAG-3′	5′-GATCTGTCCTCTTCCTTTAG -3′
ABCC4	5′-GGCAGTGACGCTGTATGG-3′	5′-CGCCAGGTCTGACAGTAAAG-3′
ABCC5	5′-AGAGGTGACCTTTGAGAACGCA-3′	5′-CTCCAGATAACTCCACCAGACGG-3′
ABCG2	5′-GCGACCTGCCAATTTCAAATG-3′	5′-GACCCTGTTAATCCGTTCGTTT-3′
NRF2	5′-CAGCGACGGAAAGAGTATGA-3′	5′-TGGGCAACCTGGGAGTAG-3′
PD-1	5′-CAGGGTGACAGAGAGAAGGG-3′	5′-CCTGGCTCCTATTGTCCCTC-3′
MT1	5′-CGTTGGTGCTGATGTCG-3′	5′-AGTTTGGGTTTGCGGTC-3′
MT2	5′-CAACTGCTGCGAGGCG-3′	5′-GGCGGTGGTGACGATG-3′
MT3	5′-GGAACCCAAGTCTTTCAACGG-3′	5′-TGGGCTCTTCCTTCCAGATGG -3′

### Human inflammation antibody array membrane (40 targets) for quantification detection of inflammatory mediators in a response to 1 mM melatonin treatment

According to Hamed et al.^15^, after HepG2 cells were seeded onto 6-well plates (300,000 cells/well), cells were treated with 1 mM melatonin for 24 h, and then lysed with RIPA lysis buffer supplemented with protease inhibitor cocktail (Invitrogen, USA) for total protein extraction. Total protein concentration was estimated using the BCA protein assay kit (Cat# 23225; Thermo scientific, USA) by following the manufacturer’s instructions. The protein concentration of each sample was calculated from the Bovine Serum Albumin (BSA) standard curve using Prism 8 (GraphPad Software Inc; San Diego, CA, United States). Human inflammation antibody array membrane (cat# ab 134003, Abcam, USA) was used for the quantification of forty inflammatory factors involved in inflammatory pathway signaling. The protocol steps of the array were followed as; after blocking the array membrane, a final sample concentration of 140 µg/ml was incubated with the blocked membrane overnight at 4 °C. Then, the membrane was incubated with 1X Biotin-Conjugated Anti-Cytokines overnight at 4˚C after excessive washing steps. Gentle washing was done with buffers I, and II provided in the kit. Later on, the membrane was incubated with diluted HRP-Conjugated Streptavidin for 2 h at RT. After washing the membrane, an equal volume of detection buffer C, and detection buffer D was mixed and then incubated with the membrane for 2 min at RT before Chemiluminescence detection, and imaging of the spots signals' by a CCD camera of chemiluminescence imager (UVP, UK). The raw densitometry data was measured with the Image Studio Lite software, (https://www.licor.com/). Then, the background signals were subtracted, and normalization to the positive control was calculated before comparing analyte-by-analyte to determine relative differences in cytokine expression in each sample.

### Statistical analysis

As our data were determined to be normally distributed after assessing their normality by the Shapiro–Wilk test, Q-Q (Quantile–Quantile) plot, and charts; the data were assumed to be parametric with equal sample sizes and variances. Accordingly, a student’s *t*-test was calculated using Microsoft Excel (version of 2007, Microsoft Corporation, USA) to detect the significant differences among the treated cells.

*P-*values of < 0.05 were considered significant (*) and *P-*values of < 0.01 were considered highly significant (**). All data were calculated as mean ± standard error of the mean (SEM). All data were reproducible, and each test was done in triplicates. GraphPad Prism version 5.0 (GraphPad Software Inc., San Diego, CA, USA) was used for statistical analyses and establishing of the figure charts.

### Ethical approval

This investigation has complete compliance with ethical standards.

## Results

### Effect of melatonin doses on HepG2 cell viability, and proliferation of cells

**Fig.1:**
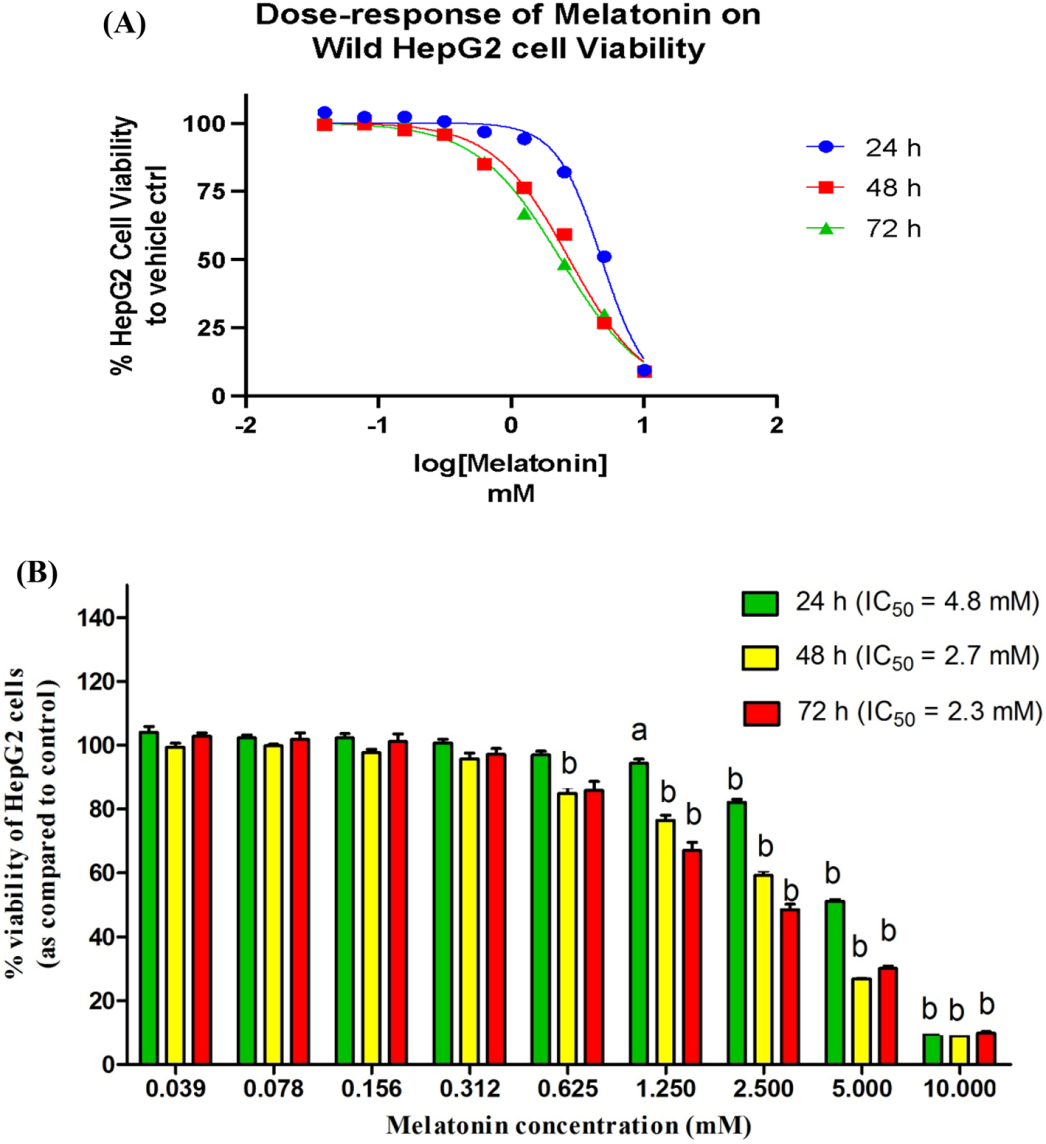
The anti-proliferative activity of melatonin toward HCC cells. (**A**) Dose–response curve shows the cytotoxic effect of melatonin on wild HepG2 cells viability using MTT assay. Wild HCC cells (HepG2) were treated with various concentrations of melatonin (0–10 mM) for 24 h, 48 h, and 72 h. Melatonin was found to reduce the % viability of HepG2 cells in a time and concentration-dependent manner. GraphPad Prism version 8.0 (non-linear regression model) was used to calculate IC_50_ values of melatonin for each time interval. The experiment was performed in octuplets. (**B**) A column chart shows the calculated IC_50_ doses of melatonin for each incubation time (24 h, 48 h, and 72 h). Data are presented as mean ± SEM. “a” represents *p* < 0.01, and “b” represents *p* < 0.001, as compared to control.

As shown in Fig. 1, various concentrations of melatonin ranged from 0 to 10 mM for 24 h, 48 h, and 72 h were found to reduce the viability of HepG2 cells in a time-concentration dependent manner. The calculated 50% inhibition concentrations (IC50) of melatonin were 4.8 mM, 2.7 mM, and 2.3 mM at 24, 48 h, and 72 h, respectively. The concentrations of melatonin used in the next experiment were selected according to the minimal anti-proliferative doses of melatonin, which decreased cell viability percentage after 24 h. Therefore, 1 mM and 2.5 mM melatonin were used to test their effects on cell cycle distribution by flow cytometry.

### Counting of accumulated cells in cell cycle phases by flow cytometry

**Figure 2:**
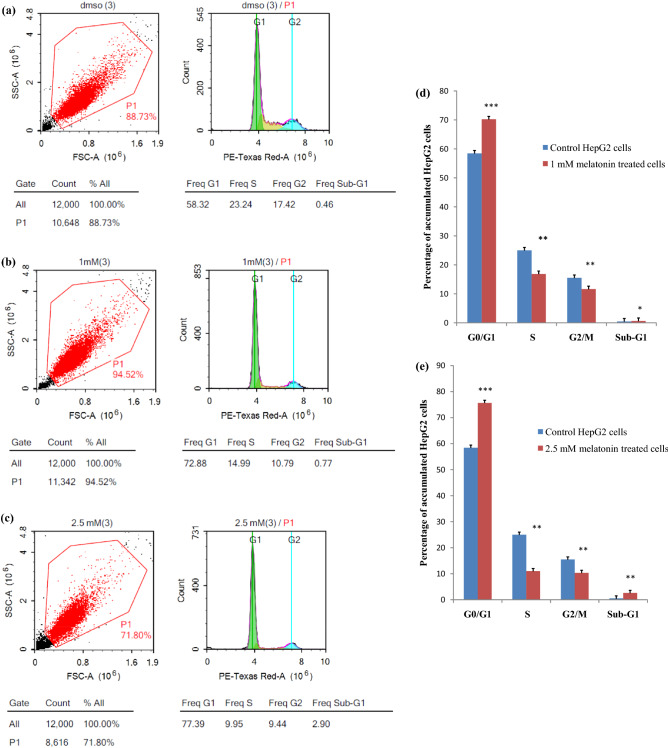
The cell cycles arrest induction by melatonin treatment. The effect of melatonin doses (1 mM, and 2.5 mM) for 24 h on HepG2 cell cycle distribution using flowcytometry is shown in representative charts (**a**) Cell cycle distribution in control vehicle untreated HepG2 cells, (**b**) cell cycle distribution in 1 mM melatonin treated HepG2 cells, (**c**) cell cycle distribution in 2.5 mM melatonin treated HepG2 cells, (**d**) the cell cycle distribution column chart of 1 mM melatonin-treated HepG2 cells, and (**e**) the cell cycle distribution column chart of 2.5 mM melatonin-treated HepG2 cells. Melatonin was detected to increase the accumulation of HepG2 cells in the G0/G1 phase of the cell cycle in percentages of 70.20% and 75.70% at melatonin concentrations of 1 mM and 2.5 mM, respectively. The experiment was repeated in triplicate. ***P < 0.001, **P < 0.01, and *P < 0.05 as compared to control.

To examine the effect of both melatonin concentrations 1 mM, and 2.5 mM on HepG2 cell cycle distribution after incubation time of 24 h. Our results determined a highly significant accumulation (*P* < 0.001) of cells in G0/G1-phase with a medium (n = 3) percentage of 70.20%, and 75.70% for 1 mM, and 2.5 mM, respectively (Fig. 2, and Table 3) in comparison to the control vehicle cells. G0/G1-phase denotes the non-proliferating cells. Compounds with anti-proliferative effects (regardless of their cytotoxicity) are expected to increase this cell population significantly^17^. Accordingly, our results confirm the antiproliferative properties of melatonin at the minimal inhibition concentration of 1 mM in comparison to 2.5 mM after 24 h incubation. Additionally, our estimation detected accumulated cells (2.62%) in a sub-G1 phase of 2.5 mM treated HepG2 cells after 24 h, and this phase represents cells with fragmented DNA that will undergo apoptosis^18^.

**Table 3:** The mean percentage of cells in each phase of the HepG2 cell cycle for each treatment of melatonin after 24 h incubation time.

	G0/G1 (%)	S (%)	G2/M (%)	Sub-G1 (%)
DMSO (control cells)	58.45	25	15.50	0.50
1 mM melatonin	70.20***	16.87**	11.66**	0.66*
2.5 mM melatonin	75.70***	11.00**	10.33**	2.62**

***P < 0.001, **P < 0.01, and *P < 0.05.

### Regulation of the expressed genes controlling apoptosis, drug efflux, anti-oxidation, and melatonin crossing in 1 mM melatonin-treated cells

**Figure 3:**
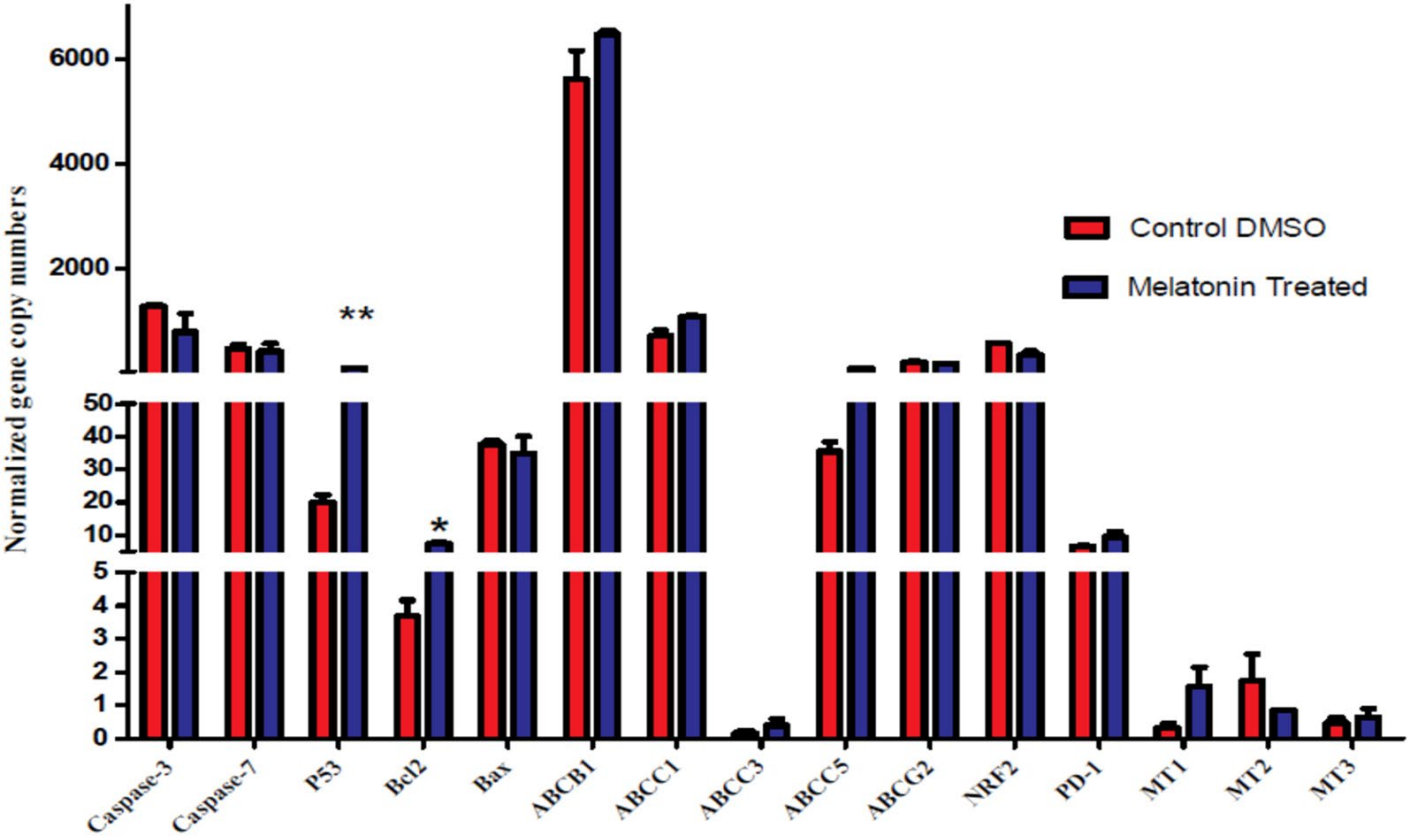
The gene expression pattern verification by qRT-PCR. The effect of 1 mM melatonin for 24 h on genes expressions of wild HepG2 cells using qRT-PCR shows a highly significant increase in the P53 gene expression level (*P* < 0.01), and a significant increase in the Bcl2 gene level (*P* < 0.05) that is associated with a slightly decreases in the expression levels of pro-apoptotic genes caspase-3, caspase-7, and BAX. Observed increases in the expression levels of genes contributed to drug resistance including *ABCB1*, *ABCC1*, and *ABCC5* are additionally shown.

Consequently to the significant antiproliferative effect of 1 mM melatonin in HepG2 cells after 24 h, as this dose was found to induce cell cycle arrest at G0/G1 phase, we preferred to choose this concentration to elucidate the effect of such minimal inhibition concentration of melatonin on the expression of some genes contributed to apoptosis, drug resistance, anti-oxidation, and melatonin entrance through the cell membranes. Our data reported a highly significant increase (*P* < 0.01) in the P53 gene expression level as compared to the control. This result proves the role of elevated expressed P53 in cell cycle arrest and accumulation of cells in the G0/G1 phase. On the other hand, we detected that the cytotoxic, and anti-proliferative action of melatonin is independent of apoptosis. As our apoptotic gene expression profiles indicated a significant increase (P < 0.05) in the expression level of the anti-apoptosis Bcl2 gene, and a slightly decrease in pro-apoptotic caspase-3, caspase-7, and Bax genes. Surprisingly, our outcomes recorded an increase in the expression levels of genes involved in drug resistance development including ABCB1, ABCC1, and ABCC5 (Fig. 3). Nevertheless, ABCG2 was found to be expressed in lower levels than the control, as for the anti-oxidation mediator NRF2 gene. These cells don't express ABCC2, and the levels of melatonin receptors᾿ expressive genes were recorded to be not significant.

### The effect of 1 mM melatonin on the expression levels of inflammatory factors involved in the inflammation pathway

**Figure 4:**
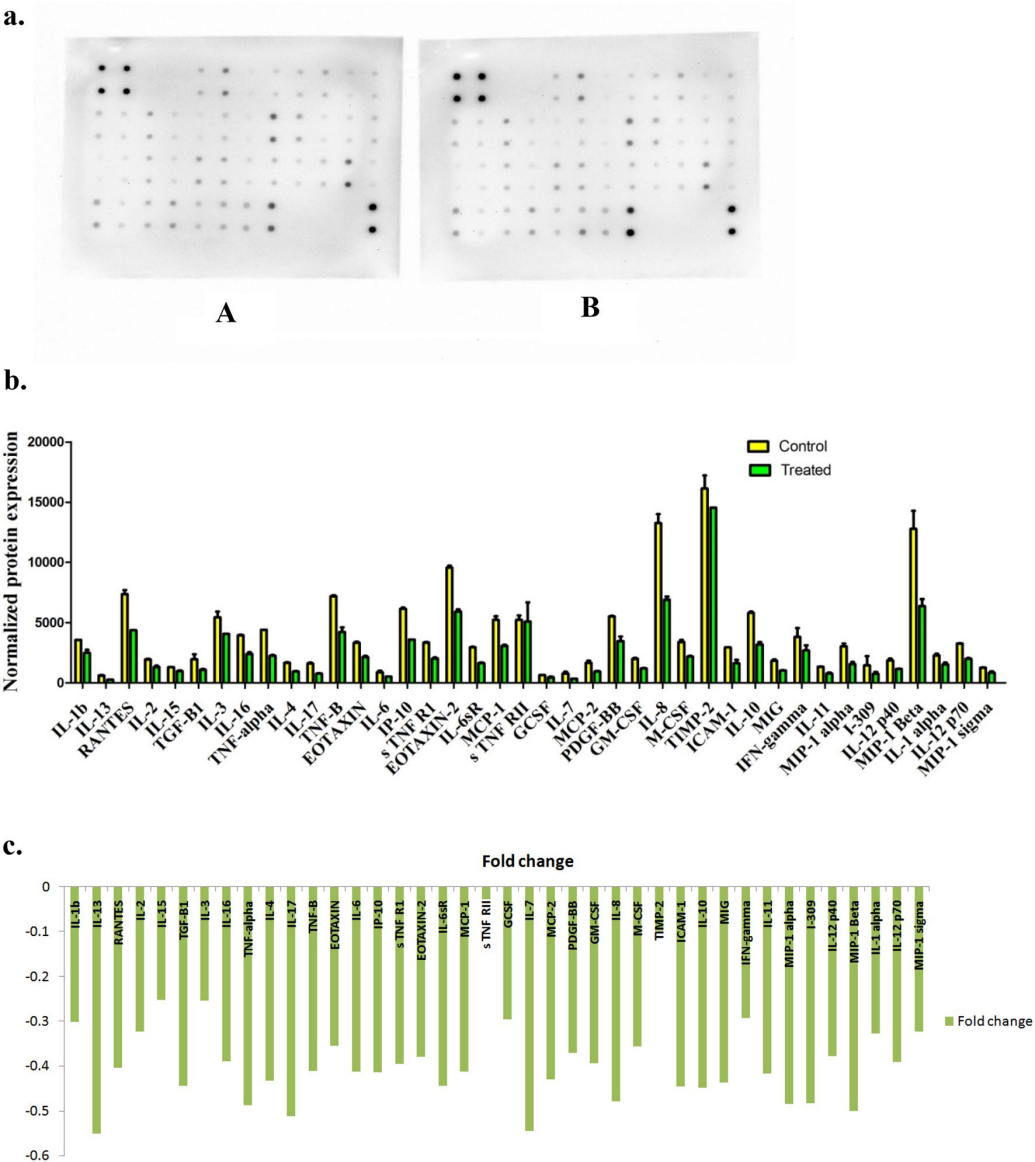
The inhibitory effect of 1 mM melatonin on the inflammation. **a**. Human inflammation antibody array membranes detect signal spots of 40 targets**/**membrane. (**A**) The array membrane of vehicle control (DMSO treated) HepG2 cells, (**B**) The array membrane of 1 mM melatonin-treated HepG2 cells for 24 h. After development of the membranes, the imaging of the spots signals' was detected by Chemiluminescence using CCD camera of chemiluminescence imager (UVP, UK), and the raw densitometry data was measured with the Image Studio Lite software, (https://www.licor.com/), **b.** The anti-inflammatory effect of 1 mM melatonin. The graph shows the effect of 1 mM melatonin for 24 h on the mean expression levels of normalized inflammatory mediators in HepG2 cells, in a comparison to the control untreated cells. After subtracting the background signals, each spot was normalized to the positive control before comparing analyte-by-analyte to determine relative differences in inflammatory factors expression for each sample. All cytokines were expressed in non-significant varied lower levels as compared to control untreated HepG2 cells. The data were calculated as a mean of duplicate ± SEM, and **c.** the calculated fold changes (of 40 inflammatory factors expressed in 1 mM melatonin-treated HCC-HepG2 cells after incubation time of 24 h) to control. The chart displays the decreasing of the expression levels of all detected inflammatory mediators with the lowest expression levels were calculated for interleukin (IL)-7, and IL-13.

As illustrated in Fig. 4a–c, our antibody array membrane results approved the anti-inflammatory effect of the minimal growth inhibition dose of melatonin, 1 mM. All inflammatory molecules **(**Table 4) were detected to be mediated in lower levels after 24 h exposure to 1 mM melatonin, as a comparison to their expression in control cells.

**Table 4:** Array map of the spots on the human inflammatory antibody array membrane (40 targets).

	A	B	C	D	E	F	G	H	I	J	K	L
1	Pos	Pos	Neg	Neg	EOTXIN	EOTAXIN-2	GCSF	GM-CSF	ICAM-1	IFN-γ	I-309	IL-1α
2	Pos	Pos	Neg	Neg	EOTAXIN	EOTAXIN-2	GCSF	GM-CSF	ICAM-1	IFN-γ	I-309	IL-1α
3	IL-1b	IL-2	IL-3	IL-4	IL-6	IL-6sR	IL-7	IL-8	IL-10	IL-11	IL-12 p40	IL-12 P70
4	IL-1b	IL-2	IL-3	IL-4	IL-6	IL-sR	IL-7	IL-8	IL-10	IL-11	IL-12 p40	IL-12 p70
5	IL-13	IL-15	IL-16	IL-17	IP-10	MCP-1	MCP-2	M-CSF	MIG	MIP-1α	MIP-1β	MIP-1δ
6	IL-13	IL-15	IL-16	IL-17	IP-10	MCP-1	MCP-2	M-CSF	MIG	MIP-1α	MIP-1β	MIP-1δ
7	RANTES	TGF-β1	TNF-α	TNF-β	S TNF RI	S TNF RII	PDGF-BB	TIMP-2	BLANK	BLANK	Neg	Pos
8	RANTES	TGF-β1	TNF-α	TNF-β	S TNF RI	S TNF RII	PDGF-BB	TIMP-2	BLANK	BLANK	Neg	Pos

## Discussion

Hepatocellular carcinoma (HCC) is the primary life-threatening liver malignancy in males, and females, globally. Additionally, it is one of the most challenging tumors to be treated. Thus, there is an emerging necessity to find alternatives or even complementary drugs that will improve the available treatment strategies. Melatonin is a natural hormone that was detected to be secreted by a few organs of the human body for the regulation of physiological processes through diverse actions. Recently, some studies have examined the antitumor activities of melatonin against several cancers. However, more investigations are required to underlie the effective functions, mediated regulatory mechanisms, and the conflicted responses of melatonin treatment in HCC^13,19^.

In the current study, HCC-in vitro wild HepG2 cells were used and treated with a scale range of melatonin doses (0–10 mM) at interval incubation times (24 h, 48 h, and 72 h). Our results indicated the decrease in % cell viability that was dependent on incubation time, and dose of melatonin, with minimal effective inhibition doses at 1 mM, and 2.5 mM after 24 h. Numerous studies were conducted in different cell types of HCC, including HepG2^2,19–22^, Hep3B^23^, HuH-7^4^, and SMMC-7721^21^, and treated with different concentrations of melatonin (10^–6^, 1, 2, 2.5 mM) at incubation times ranged from 24 to 96 h, agree with our findings. The antiproliferative effect of our administrated doses of melatonin was associated with an inhibition of the cell cycle at the G0/G1 phase, and a highly significant increase in the expression level of the P53 gene which controls the cell cycle progression. This inhibition activity was confirmed by other supportive studies in various cancers^22,24–26^. Additional studies reported cell cycle arrest at the G2/M phase^2,27^. The higher doses of melatonin could be the cause of cell cycle arrest and accumulation of cells at the G2/M phase^28^.

As a dichotomous action of 1 mM melatonin after 24 h treatment in our investigation, we recorded a significant increase in the expression level of the anti-apoptotic Bcl2 gene, besides a non-significant down-regulation of pro-apoptotic caspase-3, caspase-7, and Bax genes. Authors of previous publications were in contrast to our results, as they detected apoptotic effects for melatonin treatment that depend on the activation of caspases, and reduction of Bcl2^2,11,19,26,29,30^. However, only a few reports referred to the anti-apoptotic role of melatonin. In human hexokinase 2 (HK2) cells, an immortalized proximal tubule epithelial cell line, melatonin was found to counteract the lethal effects of cisplatin, by inducing its anti-apoptotic actions. The intermediate events in this process were observed to be mediated through the regulation of expression BAX/Bcl-2 ratio and disruption of mitochondrial membrane potential^31^. Additionally, Shen et al. supposed that pathways other than those associated with apoptosis or necrosis inhibited the proliferation of ovarian cancer cell lines (OVCAR-429 and PA-1), as they indicated non-significant increases in the percentage of cells undergoing necrosis, apoptosis or caspase 3 activation after 4 h of melatonin treatment at 400 to 800 μM^28^. The controversial behavior of this indole regarding apoptosis depends on the difference in treatment time and doses of melatonin.

Interestingly, our flow cytometric data estimated started appearing of a low percentage of cells (2.62%) in the sub-G1 phase after treatment of HepG2 cells with 2.5 mM of melatonin. The sub-G1 phase represents cells with fragmented DNA content and will undergo apoptosis^32^. Cellular arrest mediated by melatonin in this phase of the cell cycle is in favor of pro-apoptotic and anti-proliferative effects for this indole on HCC^19^. Accordingly, the cellular apoptotic induction by melatonin could be dependent on the elevated doses of this indole^28^. More research is needed to elucidate this suppose.

Although inflammation is important for cancer proliferation and metastasis^33^, multiple studies confirmed the anti-inflammatory role of melatonin treatment in various in vitro cancer models treated with 1 mM melatonin for 24 h via down-regulation of individual inflammatory mediators, such as NF-*k*B^34^, IL-1α, IL-1β, TNF-α, COX-2, IL-8 and MCP-1^9^, the present study is the first one which indicates the anti-inflammatory effect of 1 mM melatonin through targeting forty inflammatory mediated factors by using Human inflammation antibody array membrane. This inhibition could be mediated through the increased expression level of the P53 gene, as p53 has been reported to inhibit inflammatory responses, and functional loss of p53 causes excessive inflammatory reactions^15,33^.

While Colombo and the co-working team reported a decrease in the expression of NF-*k*B in breast cells after exposure to 1 mM melatonin for 24 h, they detected an increase in the same inflammatory transcription factor in HepG2 cells. So, they concluded that this double role in the expression of NF-*k*B depends on the cell type^34^.

Unfortunately, our results showed for the first time a somewhat increase in the expression of ATP-binding cassette transporters (ABCB1, ABCC1, and ABCC5) which mediate the drug efflux outside the cells and facilitate the development of drug resistance phenomena. Nevertheless, the expression of ABCG2 was quantified to be reduced. The reduction in the expression level of ABCG2 is a result of the methylation of its promoter by the action of melatonin to overcome drug resistance^35^.

In contrast to the majority of studies that approved melatonin as an anti-oxidant agent that can induce the expression of NRF2^30,36,37^. Our data reported NRF2 to be expressed at lower levels after the treatment of cells by melatonin. This cellular behavior could be explained according to the status of oxidative stress dose level. Consequently, we can predict that the unexpected action of melatonin in the present wild HCC-HepG2 cells could be due to temporary signaling of the autophagy process upon induction by melatonin^30^, which is negatively regulated by the apoptosis mechanism^38^. Despite the detected significant increase in Bcl2 expression level, it has recently doesn't found to directly affect autophagy processing^39^. Generation of ATP was reported to be associated with autophagy^40,41^, thus it is expected to estimate higher expression levels of ATP-binding cassette transporters^42^.

We obtained through our experiments that, the mediated actions of melatonin were independent of melatonin receptors (MT1, MT2, and MT3) which were found to be insignificantly expressed upon induction with 1 mM melatonin for 24 h. On the other hand, studies^13,27^ suggested that the growth and migration suppressive activity of 1 mM, 2 mM, and 2.5 mM doses of melatonin could be mediated through the expression induction of different levels of MT1/MT2/MT3 receptors in Huh7 and HepG2 cell lines after incubation times ranged from 2 to 6 days. However, Wang et al.^13^ recommended further studies to identify the specific receptors through which melatonin mediates its antitumor activities. The induction of melatonin receptors depends on the treatment time and the melatonin dose administered to the HepG2 tumor cells^27^. In addition, studies conducted in breast, and colon cancers stated that the low sensitivity to melatonin treatments of 1 mM, and 2 mM was due to the low expressing levels of MT1/MT2 receptors^43,44^.

Melatonin is an effective anti-proliferative drug that can inhibit cell cycle progression in a mechanism that is independent of apoptosis. Moreover, melatonin is a promising candidate for targeting the inflammatory pathway. In conclusion, Melatonin could be administrated individually or in combination with other chemotherapies to introduce a better treatment strategy for HCC patients, but its consumption should be cautious to avoid the controversial responses of the treatment. The controversial effect of melatonin could be mediated through other alternative types of programmed cell death such as autophagy. As a result of ATP production, which is associated with autophagy, there was an increase in the ATP- binding cassette transporters (ABCs).

## Data Availability

All are available in the current research paper.
